# The British Association

**Published:** 1898-09-17

**Authors:** 


### THE BRITISH ASSOCIATION.

The presidential address of Sir William Crookes, at the annual meeting of the British Association for the Advancement of Science, differed widely from most of those which have been delivered by his predecessors. The part of the address which will chiefly appeal to a large section of the public is its conclusion, of the fitness of which it is evident that Sir William himself felt some doubt. In this he dealt with the question of the so-called "psychic research," to which, as is well known, much of his time and thoughts have been devoted. Thirty years ago he published an account of experiments tending (in his opinion) to show that outside our scientific knowledge there exists a force exercised by intelligence differing from the ordinary intelligence common to mortals. To this opinion he still adheres, but if he were now for the first time introducing such inquiries to the world of science, he would choose a starting-point different from that of old. He would begin with telepathy, with the fundamental law, as he believes it to be, that thoughts and images may be transferred from one mind to another without the agency of recognised organs of sense; that knowledge may enter the human mind without being communicated in any hitherto known or recognised way. The word "telepathy," however, is an unfortunate one. It was coined in order to denote the influence of a remedy applied at a distance from the disease—the supposed influence, for example, of a salve applied to the weapon with which a wound had been inflicted; and the element of "disease" has no essential place in Sir William Crookes' hypothesis. The word has, indeed, been wrested from its original signification to describe nearly all that mesmerists and their congeners have included under the term "clairvoyance," and it has become deeply and beyond recovery ear-marked as belonging to the vocabulary of fraud. It seems to us, then, that Sir William Crookes' dalliance with it, and with much that it connotes, is in the highest degree unfortunate and to be deplored. The whole subject is of infinite attractiveness to many minds of a very feeble and disordered type, minds which have never acquired even the first principles of scientific investigation, and which are utterly unable to resist the temptation of believing any statement they would like to be true. Nevertheless, if such matters are to be thought fitting for discussion at a meeting of the British Association for the Advancement of Science, we cannot complain of the manner in which it was introduced. Telepathy—if such a thing exists—has so often been assumed to involve something of the supernatural that it is very satisfactory to find Sir William Crookes so definitely pinning it down as a manifestation of physical force. Many, no doubt, there are whose main interest in what is called "psychical research" is the hope that through it evidence will be forthcoming of an ever-present spirit world around us. Sir William, however, knocks all such ideas on the head when he says, "If telepathy take place we have two physical facts — the physical change in the brain of A, the suggester; and the analogous physical change in the brain of B, the recipient of the suggestion. Between these two physical events there must exist a train of physical causes." This states the matter with scientific precision, and while it will, we hope, damp the ardour of those who take every instance of apparent thought-transference as one more proof of ghostly interference in mundane affairs, it will, on the other hand, discourage the tendency, which has of late been perhaps too much in evidence among materialistic thinkers, to throw doubt upon all phenomena which do not admit of immediate explanations according to the accepted canons of physical science. Heat, light, and electricity are no doubt good, respectable modes in which energy can be made manifest, but we have no right to deny the possibility of its being transmitted in a score of other ways—except, indeed that we have not observed them—and it must be noted that the "ether," by means of which we explain so much in physics, might, perhaps, also explain much more which at present we are apt to look upon with great suspicion. In any case, however, it is most desirable that the investigation of strange mental phenomena should be treated as a matter of science, and should be disassociated from that hunt for the supernatural which to many is so pleasing a pursuit. We are far from disputing the importance of the problems which Sir William Crookes is endeavouring to solve, or the possibility that their solution may be arrived at upon the lines which he is following, and following, we are sure, with a caution and a sagacity not always to be expected from his associates. There is a fine passage in which Faraday tells us how little the world knows of the many thoughts and theories which pass through the mind of a scientific investigator, only to be crushed in silence and secrecy by his own severe criticism and adverse examination; and how, in the most successful instances, not a tenth of the suggestions, the hopes, the preliminary conclusions have been realised. We could have wished that Sir William's hopes, on a subject so deeply defiled by the arts of the impostor, had been locked within his own breast until the arrival of a time at which he could speak of discoveries in the language, not only of scientific precision, but in that of scientific certainty.

